# Interaction of Serum Alkaline Phosphatase and Folic Acid Treatment on Chronic Kidney Disease Progression in Treated Hypertensive Adults

**DOI:** 10.3389/fphar.2021.753803

**Published:** 2022-01-13

**Authors:** Yuanyuan Zhang, Panpan He, Guobao Wang, Min Liang, Di Xie, Jing Nie, Chengzhang Liu, Yun Song, Lishun Liu, Binyan Wang, Jianping Li, Yan Zhang, Xiaobin Wang, Yong Huo, Fan Fan Hou, Xiping Xu, Xianhui Qin

**Affiliations:** ^1^ State Key Laboratory of Organ Failure Research, Guangzhou Regenerative Medicine and Health Guangdong Laboratory, Division of Nephrology, Nanfang Hospital, National Clinical Research Center for Kidney Disease, Guangdong Provincial Institute of Nephrology, Guangdong Provincial Clinical Research Center for Kidney Disease, Guangdong Provincial Key Laboratory of Renal Failure Research, Southern Medical University, Guangzhou, China; ^2^ Institute of Biomedicine, Anhui Medical University, Hefei, China; ^3^ Beijing Advanced Innovation Center for Food Nutrition and Human Health, College of Food Science and Nutritional Engineering, China Agricultural University, Beijing, China; ^4^ Department of Cardiology, Peking University First Hospital, Beijing, China; ^5^ Department of Population, Family and Reproductive Health, Johns Hopkins University Bloomberg School of Public Health, Baltimore, MD, United States

**Keywords:** serum alkaline phosphatase, folic acid, CKD progression, hypertension, cohort study

## Abstract

The relation of alkaline phosphatase (ALP) with chronic kidney disease (CKD) is still uncertain. We aimed to examine the prospective association between serum ALP and CKD progression, and the modifying effect of serum ALP on folic acid in preventing CKD progression in treated hypertensive patients. This is a post-hoc analysis of 12,734 hypertensive adults with relevant measurements and without liver disease at baseline from the renal sub-study of the China Stroke Primary Prevention Trial, where participants were randomly assigned to daily treatments of 10 mg enalapril and 0.8 mg folic acid, or 10 mg enalapril alone. The primary outcome was CKD progression, defined as a decrease in estimated glomerular filtration rate (eGFR) of ≥30% and to a level of <60 ml/min/1.73 m^2^ if baseline eGFR was ≥60 ml/min/1.73 m^2^; or a decrease in eGFR of ≥50% if baseline eGFR was <60 ml/min/1.73 m^2^; or end-stage renal disease. Over a median of 4.4 years, in the enalapril only group, participants with baseline serum ALP≥110IU/L (quartile 4) had a significantly higher risk of CKD progression (3.4% *vs* 2.3%; adjusted OR,1.61; 95%CI:1.11, 2.32), compared with those with ALP<110IU/L. For those with enalapril and folic acid treatment, compared with the enalapril only treatment, the risk of CKD progression was reduced from 3.4 to 2.1% (adjusted OR, 0.53; 95%CI:0.34, 0.83) among participants with baseline ALP≥110IU/L, whereas there was no significant effect among those with ALP<110IU/L. In hypertensive patients, higher serum ALP was associated with increased risk of CKD progression, and this risk was reduced by 47% with folic acid treatment.

## Introduction

Chronic kidney disease (CKD) is a global public health problem, affecting more than 500 million people worldwide (Chen et al., 2019). CKD is independently associated with end-stage renal disease (ESRD), cardiovascular disease (CVD), and all-cause mortality (Go et al., 2004; Coresh et al., 2014; Tanaka et al., 2017), and leads to high health care costs (Jha et al., 2013). It is therefore important to identify more risk factors of CKD that would reduce the public health burden and serious clinical consequences by leading to early detection and prevention.

Previous studies have found an obvious association between liver and renal disease, and suggested that liver damage and CKD may share some common mechanisms, such as oxidative stress and inflammation (Contreras et al., 2007; Er et al., 2020; Sansoè et al., 2020). Alkaline phosphatase (ALP) is a generally accepted clinical useful marker for liver and bone disease (Harmey et al., 2004). It has been reported that ALP is an important risk factor for cardiovascular diseases (CVD) and mortality (Park et al., 2013; Kunutsor et al., 2014; Kabootari et al., 2018), owing to its role in endothelial dysfunction, inflammation, and oxidative stress (Haarhaus et al., 2017). Moreover, the serum ALP levels are commonly elevated in CKD and dialysis patients (Damera et al., 2011). Due to the similar involved mechanisms and the increased ALP levels in CKD patients, we speculate that ALP may also play an important role in the development of CKD. However, few prospective studies have examined the possible relation of ALP and the development of CKD in the general population.

Furthermore, in a previous study, we reported that folic acid treatment can delay the progression of CKD by 55% among hypertensive patients with CKD (Xu et al., 2016). Given that folic acid, in addition to having antioxidant and anti-inflammatory properties, can directly improve endothelial function (Title et al., 2000; Joshi et al., 2001; Solini et al., 2006; Spence et al., 2017), it is biologically plausible that folic acid treatment may counteract the possible detrimental effects of elevated ALP on CKD. However, this hypothesis has not been tested in previous studies.

Our current report was motivated by the limited data regarding the ALP and incident CKD, and an exceptional opportunity to address this question in a large, randomized controlled folic acid intervention trial with regular antihypertensive treatments, BP measurements and CKD status reports. Specifically, using data from the renal sub-study of the China Stroke Primary Prevention Trial (CSPPT) (Xu et al., 2016), we sought to investigate the effect of serum ALP on the risk of CKD progression and on the efficacy of folic acid treatment in prevention of CKD progression in general hypertensive adults.

## Materials and Methods

### Study Design and Participants

Details regarding the study design, methods, and major results of the CSPPT (ClinicalTrials.gov identifier NCT00794885) (Qin et al., 2013; Huo et al., 2015; Qin et al., 2017a; Qin et al., 2017b) and the renal sub-study of the CSPPT (Xu et al., 2016) have been reported previously. Briefly, the CSPPT was a multi-community, randomized, double-blind, controlled trial conducted from May 2008 to August 2013 in 32 communities in Anhui and Jiangsu provinces in China. Eligible participants were men and women aged 45–75 years who had hypertension, defined as seated, resting systolic blood pressure (SBP) ≥140 mmHg or diastolic blood pressure (DBP) ≥90 mmHg at both the screening and recruitment visit, or who were on anti-hypertensive medication. The major exclusion criteria included history of physician-diagnosed stroke, myocardial infarction, heart failure, post-coronary revascularization, and/or congenital heart disease, and/or current supplementation by folic acid, vitamin B12 or vitamin B6.

In the CSPPT, a total of 20,702 eligible participants were enrolled. The renal sub-study of the CSPPT included 15,104 participants from 20 communities in Jiangsu province with an estimated glomerular filtration rate (eGFR) ≥30 ml/min/1.73 m^2^. Our current study is a post-hoc analysis of the renal sub-study of the CSPPT, where a total of 12,734 participants with complete relevant measurements and without liver disease (self-reported chronic hepatitis, hepatic adipose infiltration, or cirrhosis) at baseline were included (Supplemental Figure S1).

The parent study (the CSPPT) and the current study were approved by the Ethics Committee of the Institute of Biomedicine, Anhui Medical University, Hefei, China (FWA assurance number: FWA00001263). All participants provided written informed consent.

### Intervention and Follow-Up

Eligible participants were randomized to receive a daily oral dose of one tablet containing 10-mg enalapril and 0.8-mg folic acid (single pill combination, the enalapril-folic acid group) or one tablet containing 10-mg enalapril only (the enalapril-only group).

Participants were scheduled for follow-up every 3 months. At each follow-up visit, BP was measured; study drug compliance, concomitant medication use, adverse events and possible endpoint events were documented by trained research staff and physicians.

### Laboratory Assessment

Serum and spot urine samples were obtained from each study participant at baseline and at the exit visit. Serum fasting creatinine, ALP, gamma glutamyl transpeptidase (GGT), alanine aminotransferase (ALT), aspartate aminotransferase (AST), total homocysteine (tHcy), uric acid, phosphate, calcium, albumin, lipids and fasting glucose were measured with the use of automatic clinical analyzers (Beckman Coulter) at the core laboratory of the National Clinical Research Center for Kidney Disease, Nanfang Hospital, Guangzhou, China. Estimated GFR was calculated using the Chronic Kidney Disease Epidemiology Collaboration (CKD-EPI) equation (Levey et al., 2009).

### Study Outcomes

The primary outcome was CKD progression, defined as a decrease in eGFR ≥30% and to a level <60 ml/min/1.73 m^2^ at the exit visit if baseline eGFR was ≥60 ml/min/1.73 m^2^; or a decrease in eGFR ≥50% at the exit visit if baseline eGFR was <60 ml/min/1.73 m^2^; or end-stage renal disease (eGFR <15 ml/min/1.73 m^2^ or need for dialysis).

Secondary outcomes included the following: 1) CKD incidence, defined as eGFR ≥60 ml/min/1.73 m^2^ at baseline, and eGFR <60 ml/min/1.73 m^2^ at the exit visit; 2) rapid decline in renal function, defined as a decline in eGFR of 40% or more; 3) annual rate of relative decline in eGFR, estimated as ( $1-\sqrt[{t}]{\frac{eGFR\;at\;exit}{eGFR\;at\;baseline}}$ ) × 100%, where *t* is time in years from baseline to the exit visit.

### Statistical Analysis

Baseline characteristics are presented as means ± standard deviations (SDs) and proportions for continuous and categorical variables, respectively. Statistical significance of differences in baseline characteristics was assessed by baseline serum ALP quartiles using *ANOVA* tests or chi-square tests, accordingly.

Logistic (primary outcome and secondary outcome 1–2) or linear regression (secondary outcome 3) models were performed to estimate the relation of baseline serum ALP levels with the primary and secondary renal outcomes in the enalapril-only group without and with adjustment for major covariates. In addition, the effect modification of baseline serum ALP levels on folic acid efficacy in the primary outcome in the total population (participants from the enalapril-only group and the enalapril-folic acid group) were evaluated both before and after adjustment for major covariates. In addition, the association of folic acid treatment with primary and secondary outcomes across each serum ALP subgroup [≥110 (quartile 4) or <110 IU/L] were estimated and their interactions were assessed.

A two-tailed *p* < 0.05 was considered statistically significant in all analyses. R software, version 3.6.3 (http://www.R-project.org/) was used to perform all statistical analyses.

## Results

### Study Participants and Baseline Characteristics

In this study, a total of 12,734 participants (6,389 in the enalapril-only group and 6,345 in the enalapril-folic acid group) with baseline ALP measurements and complete renal outcomes, and without liver disease at baseline, were included in the final analyses (Supplemental Figure S1).

Baseline characteristics of participants in the enalapril-only group by baseline ALP quartiles are presented in Supplemental Table S1. The mean and median serum ALP levels in the enalapril-only group were 95.4 (SD, 28.2) and 92 IU/L, respectively. Participants with higher ALP levels were more likely to be female. Among females, those with higher ALP levels were more likely to be older, had higher SBP, triglycerides (TG), fasting glucose (FG), albumin-corrected calcium and phosphate levels, and a higher prevalence of diabetes and proteinuria; had a lower frequency in use of antihypertensive drugs at baseline, as well as higher time-averaged SBP during the treatment period (Supplemental Table S2). Similar trends were found in males (Supplemental Table S3).

However, almost all population characteristics at baseline, as well as time-averaged BP and concomitant medication usage during the trial were comparable between the 2 treatment groups within each baseline ALP stratum [≥110 (quartile 4) or <110 IU/L] (Table 1).

**TABLE 1:** Characteristics of the study participants by baseline serum ALP strata (<110 and ≥110 IU/L) and treatment group^a^.

Variables	ALP <110 IU/L		ALP ≥110 IU/L	
	Enalapril-only	Enalapril-folic acid	Enalapril-only	Enalapril-folic acid
Baseline	—	—	—	—
N	4727	4657	1662	1688
Male, No. (%)	2079 (44.0)	2003 (43.0)	402 (24.2)	390 (23.1)
Age, y	59.3 ± 7.8	59.2 ± 7.6	60.3 ± 6.7	60.7 ± 6.6
Body mass index, kg/m^2^	25.7 ± 3.5	25.7 ± 3.5	25.6 ± 3.5	25.9 ± 3.7
SBP at baseline, mmHg	168.0 ± 21.1	167.6 ± 20.4	170.5 ± 21.0	170.4 ± 21.4
DBP at baseline, mmHg	95.4 ± 12.0	95.4 ± 11.8	94.8 ± 12.2	95.0 ± 11.5
Current smoking, No. (%)	1153 (24.4)	1123 (24.1)	263 (15.8)	262 (15.5)
Current alcohol drinking, No. (%)	1256 (26.6)	1192 (25.6)	215 (12.9)	204 (12.1)
Diabetes, No. (%)	576 (12.2)	564 (12.1)	308 (18.5)	280 (16.6)
Proteinuria, No. (%)	406 (8.9)	427 (9.5)	191 (12.0)	190 (11.7)
Laboratory results	—	—	—	—
Total cholesterol, mmol/L	5.7 ± 1.2	5.7 ± 1.2	5.7 ± 1.3	5.8 ± 1.2
HDL-C, mmol/L	1.3 ± 0.4	1.3 ± 0.4	1.4 ± 0.4	1.4 ± 0.4
Triglycerides, mmol/L	1.7 ± 0.9	1.7 ± 1.7	1.9 ± 1.0	1.9 ± 1.0
Fasting glucose, mmol/L	6.0 ± 1.5	5.9 ± 1.5	6.4 ± 2.4	6.2 ± 2.1
Uric acid, μmol/L	295.2 ± 78.4	294.9 ± 79.8	289.0 ± 78.2	288.9 ± 77.7
Phosphate, mmol/L	1.3 ± 0.2	1.3 ± 0.2	1.4 ± 0.3	1.4 ± 0.3
Albumin-corrected calcium, mmol/L	2.4 ± 0.2	2.4 ± 0.2	2.5 ± 0.2	2.5 ± 0.2
eGFR, mL/min1.73/m^2^	93.9 ± 12.7	93.9 ± 12.8	94.9 ± 12.5	93.9 ± 13.0
Folate, ng/mL	7.6 ± 3.2	7.6 ± 3.2	7.9 ± 3.3	7.8 ± 3.3
Vitamin B12, pg/mL	406.7 ± 159.1	402.2 ± 144.7	410.6 ± 186.5	401.9 ± 161.6
Medication use, No. (%)	—	—	—	—
Antihypertensive drugs	2413 (51.0)	2363 (50.7)	794 (47.8)	787 (46.6)
Glucose-lowering drugs	71 (1.5)	92 (2.0)	34 (2.0)	36 (2.1)
Lipid-lowering drugs	45 (1.0)	43 (0.9)	11 (0.7)	13 (0.8)
Antiplatelet drugs	216 (4.6)	175 (3.8)	53 (3.2)	54 (3.2)
During treatment period	—	—	—	—
Time-averaged on-treatment SBP	139.5 ± 11.0	139.1 ± 10.6	140.1 ± 10.9	140.2 ± 11.6
Time-averaged on-treatment DBP	83.7 ± 7.3	83.7 ± 7.1	82.9 ± 7.5	82.8 ± 7.1
Medication use^b^, No. (%)	—	—	—	—
Calcium channel blockers	3837 (81.2)	3786 (81.3)	1356 (81.6)	1391 (82.4)
Diuretics	2881 (60.9)	2813 (60.4)	1005 (60.5)	1085 (64.3)
Glucose-lowering drugs	74 (1.6)	70 (1.5)	39 (2.3)	33 (2.0)
Lipid-lowering drugs	5 (0.1)	6 (0.1)	0 (0.0)	4 (0.2)
Antiplatelet drugs	50 (1.1)	39 (0.8)	9 (0.5)	14 (0.8)

a Variables are presented as Mean ± SD, or n (%).

b Regular concomitant medication was defined as 180 or more cumulative days of taking the drug of interest.

Abbreviations: ALP, alkaline phosphatase; DBP, diastolic blood pressure; eGFR, estimated glomerular filtration rate; HDL-C, high-density lipoprotein; SBP, systolic blood pressure.

### Baseline Serum Alkaline Phosphatase and the Risk of Renal Outcomes in the Enalapril-Only Group

**FIGURE 1:**
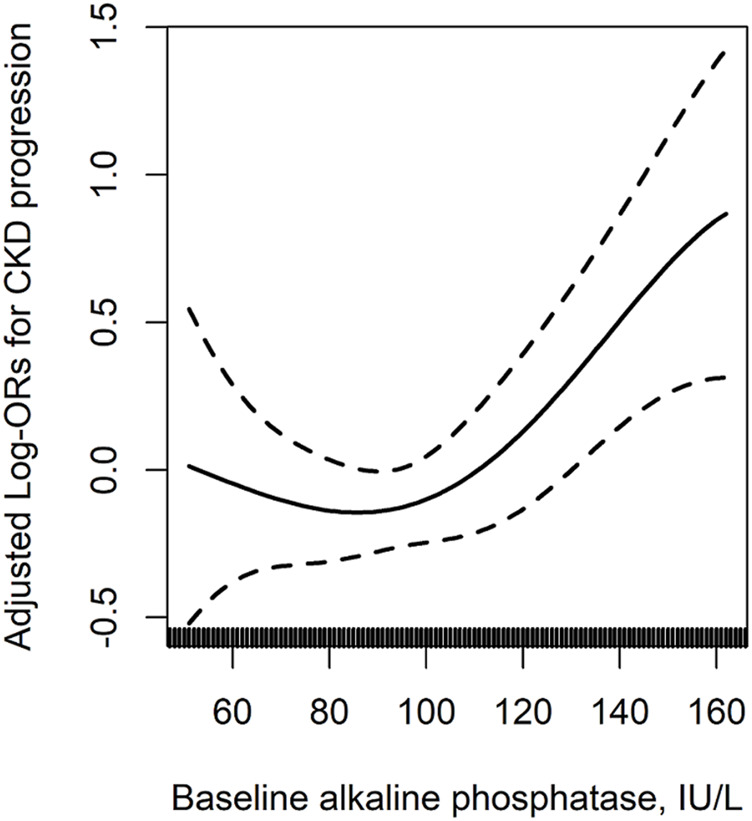
The relation of baseline serum alkaline phosphatase with CKD progression in the enalapril-only population^*^. ^*^Adjusted for age, sex, body mass index, smoking, alcohol drinking, albumin-corrected calcium, phosphate, uric acid, total cholesterol, fasting glucose, eGFR, systolic blood pressure (SBP), proteinuria and antihypertensive drug usage at baseline, as well as time-averaged SBP, the use of calcium channel blockers (CCB) and diuretics during the treatment period. ^†^Only participants with baseline eGFR ≥60 ml/min/1.73 m^2^ were included in this analysis. AbbreviationsCKD, chronic kidney disease; eGFR, estimated glomerular filtration rate.

The median follow-up duration was 4.4 years (interquartile range, 4.0–4.8 years). The relationship of serum ALP with the risk of CKD progression (Figure 1), CKD incidence (Supplemental Figure S2A), rapid decline in renal function (Supplemental Figure S2B) and annual rate of relative decline in eGFR (Supplemental Figure S2C) in the enalapril-only group are presented in Figure 1 and Supplemental Figure S2. When serum ALP was assessed as quartiles, among the enalapril-only group, a significantly higher risk of CKD progression was found in participants in quartile 4 (ALP ≥110 IU/L; 3.4% *vs* 2.3%; adjusted OR, 1.61; 95% CI: 1.11, 2.32), compared with those in quartile 1–3 (ALP <110 IU/L) (Table 2 **)**. Similar trends were found for CKD incidence (ALP ≥110 *vs* <110 IU/L; adjusted OR, 1.54; 95% CI: 1.11, 2.13), rapid decline in renal function (ALP ≥110 *vs* <110 IU/L; adjusted OR, 1.39; 95% CI: 0.88, 2.22) and annual rate of relative decline in eGFR (ALP ≥110 *vs* <110 IU/L; adjusted β, 0.24; 95% CI: 0.04, 0.44) (Table 2).

**TABLE 2:** The association between baseline serum alkaline phosphatase and renal outcomes in the enalapril-only treatment group.

ALP, IU/L	N	No. of events (%)	Crude model	Adjusted model^a^
			OR (95% CI)	OR (95% CI)
CKD progression	—	—	—	—
Quartiles	—	—	—	—
Q1 (<76)	1520	32 (2.1)	*Ref*	*Ref*
Q2 (76-<92)	1671	37 (2.2)	1.05 (0.65, 1.70)	1.04 (0.63, 1.73)
Q3 (92-<110)	1536	38 (2.5)	1.18 (0.73, 1.90)	0.96 (0.57, 1.62)
Q4 (≥110)	1662	57 (3.4)	1.65 (1.07, 2.56)	1.60 (0.97, 2.65)
Categories	—	—	—	—
Q1-3 (<110)	4727	107 (2.3)	*Ref*	*Ref*
Q4 (≥110)	1662	57 (3.4)	1.53 (1.11, 2.13)	1.61 (1.11, 2.32)
CKD incidence^b^	—	—	—	—
Categories	—	—	—	—
Q1-3 (<110)	4644	166 (3.6)	*Ref*	*Ref*
Q4 (≥110)	1636	76 (4.6)	1.31 (1.00, 1.73)	1.54 (1.11, 2.13)
Rapid decline in renal function	—	—	—	—
Categories	—	—	—	—
Q1-3 (<110)	4726	67 (1.4)	*Ref*	*Ref*
Q4 (≥110)	1662	33 (2.0)	1.41 (0.92, 2.15)	1.39 (0.88, 2.22)
	**N**	**Mean ± SD**	**β (95% CI)**	**β (95% CI)**
Decline in eGFR, % per y	—	—	—	—
Categories	—	—	—	—
Q1-3 (<110)	4726	1.3 ± 3.4	*Ref*	*Ref*
Q4 (≥110)	1662	1.7 ± 3.7	0.44 (0.25, 0.63)	0.24 (0.04, 0.44)

a Adjusted for age, sex, body mass index, smoking, alcohol drinking, albumin-corrected calcium, phosphate, uric acid, total cholesterol, fasting glucose, eGFR, systolic blood pressure (SBP), proteinuria and antihypertensive drug usage at baseline, as well as time-averaged SBP, the use of calcium channel blockers (CCB) and diuretics during the treatment period.

b Only participants with baseline eGFR ≥60 ml/min/1.73 m^2^ were included in this analysis.

Abbreviations: ALP, alkaline phosphatase; CI, confidence interval; CKD, chronic kidney disease; eGFR, estimated glomerular filtration rate; OR, odds ratio; SD, standard deviation.

In addition, similar results were also found in participants with a normal range of baseline serum ALP (20–140 IU/L) levels (Sharma et al., 2014) in the enalapril-only group (Supplemental Table S4). More importantly, further adjustment for other liver enzymes, including GGT, ALT, AST did not substantially change the results (ALP ≥110 *vs* <110 IU/L; adjusted OR, 1.67; 95% CI: 1.16, 2.42) (Supplemental Table S5).

### Effect of Baseline Serum ALP Levels on the Efficacy of Folic Acid Treatment in Preventing Renal Outcomes Among the Total Population

Among the total participants, compared with the enalapril-only group, for those with folic acid treatment, the risk of CKD progression was reduced from 3.4 to 2.1% (adjusted OR, 0.53; 95%CI: 0.34, 0.83) among participants with baseline serum ALP ≥110 IU/L. In contrast, folic acid treatment had no significant effect on CKD progression in those with baseline serum ALP <110 IU/L (2.0% in the enalapril-folic acid group *vs* 2.3% in the enalapril-only group; adjusted OR, 0.91; 95% CI: 0.68, 1.22). The interaction between ALP levels and folic acid treatment on CKD progression was significant (*p* = 0.047) (Table 3).

**TABLE 3:** Effect modification of baseline serum alkaline phosphatase levels (<110 and ≥110 IU/L) on efficacy of enalapril-folic acid treatment in renal outcomes, compared to enalapril-only treatment group.

ALP (IU/L)		Enalapril-only	Enalapril-folic acid	Crude Model		Adjusted Model^a^	
		Events (%)	Events (%)	OR (95%CI)	*P*-interaction	OR (95%CI)	*P*- interaction
CKD progression		—	—	—	0.162	—	0.047
<110		107 (2.3)	93 (2.0)	0.88 (0.66, 1.17)	—	0.91 (0.68, 1.22)	—
≥110		57 (3.4)	36 (2.1)	0.61 (0.40, 0.94)	—	0.53 (0.34, 0.83)	—
CKD incidence^b^		—	—	—	0.234	—	0.043
<110		166 (3.6)	152 (3.3)	0.93 (0.74, 1.16)	—	0.98 (0.76, 1.25)	—
≥110		76 (4.6)	56 (3.4)	0.72 (0.51, 1.03)	—	0.61 (0.41, 0.90)	—
Rapid decline in renal function				—	0.314	—	0.208
<110	67 (1.4)		60 (1.3)	0.91 (0.64, 1.29)	—	0.95 (0.66, 1.36)	—
≥110	33 (2.0)		22 (1.3)	0.65 (0.38, 1.12)	—	0.62 (0.35, 1.08)	—
	**Mean ± SD**		**Mean ± SD**	**β (95% CI)**	***P*-interaction**	**β (95% CI)**	***P*-interaction**
Decline in eGFR, % per y				—	0.059	—	0.038
<110	1.3 ± 3.4		1.2 ± 3.2	-0.07 (-0.21, 0.07)	—	-0.05 (-0.19, 0.08)	—
≥110	1.7 ± 3.7		1.4 ± 3.7	-0.33 (-0.56, -0.10)	—	-0.33 (-0.55, -0.10)	—

a Adjusted for age, sex, body mass index, smoking, alcohol drinking, albumin-corrected calcium, phosphate, uric acid, total cholesterol, fasting glucose, eGFR, systolic blood pressure (SBP), proteinuria and antihypertensive drug usage at baseline, as well as time-averaged SBP, the use of calcium channel blockers (CCB) and diuretics during the treatment period.

b Only participants with baseline eGFR ≥60 ml/min/1.73 m^2^ were included in this analysis.

Abbreviations: ALP, alkaline phosphatase; CI, confidence interval; CKD, chronic kidney disease; eGFR, estimated glomerular filtration rate; OR, odds ratio; SD, standard deviation.

Similar results were found for CKD incidence, rapid decline in renal function, and annual rate of relative decline in eGFR (Table 3), and in participants with a normal range of serum ALP levels (Supplemental Table S6). Further adjustment for other liver enzymes, (Supplemental Table S7) did not substantially change the results.

### Stratified Analyses

In all the subgroups, including sex (male *vs* female), age (<65 *vs* ≥65 years), BMI (<24 *vs* ≥24 kg/m^2^), SBP (<160 *vs* ≥160 mmHg), TC (<5.2 *vs* ≥5.2 mmol/L), tHcy [<12.5 (median) *vs* ≥12.5 μmol/L], folate [<7.3 (median) *vs* ≥7.3 ng/ml], vitamin B12 [<370.8 (median) *vs* ≥370.8 pg/ml], phosphate [<1.3 (median) *vs* ≥1.3 mmol/L], albumin-corrected calcium [<2.4 (median) *vs* ≥2.4 mmol/L], FG (<5.6 *vs* 5.6-<7.0 mmol/L *vs* diabetes), and CKD (no *vs* yes) at baseline, as well as time-averaged SBP (<140 *vs* ≥140 mmHg), diuretics usage (no *vs* yes) and calcium channel blockers usage (no *vs* yes) over the trial period, there was a greater beneficial effect of folic acid treatment for participants with higher baseline serum ALP (≥110 IU/L) than for those with lower baseline serum ALP (<110 IU/L) (Supplemental Table S8).

Of note, among those with both CKD and higher serum ALP (≥110 IU/L) at baseline, compared with enalapril alone, enalapril and folic acid treatment was associated with 73% reduction in CKD progression (3.3% in the enalapril-folic acid group *vs* 10.6% in the enalapril-only group; adjusted OR, 0.27; 95% CI: 0.11, 0.65) (Supplemental Table S8).

## Discussion

To our knowledge, this is the first study to examine the prospective association between serum ALP and CKD progression, and the modifying effect of serum ALP on folic acid in preventing CKD progression in treated hypertensive patients. We found that among hypertensive adults, those participants with higher serum ALP had significantly increased risk of CKD progression. More importantly, in participants with higher serum ALP, folic acid treatment significantly reduced the risk of CKD progression by 47 and 73%, respectively, in the total population and in those with CKD at baseline. Our findings are clinically meaningful in term of the magnitude of the CKD progression risk reduction.

Multiple previous studies have reported that ALP is associated with increased risk of mortality in CKD patients, despite whether they were dialysis-dependent (Fan et al., 2017; Zhan et al., 2019) or non-dialysis dependent patient (Taliercio et al., 2013; Sumida et al., 2018). To date, however, only two small studies (Yamazoe et al., 2016; Majoni et al., 2020) have investigated the association between serum ALP and renal outcomes. One study conducted in Japan (Yamazoe et al., 2016), reported that the risk of worsening renal function during hospitalization increased by 69 and 95%, respectively, in patients in ALP tertile 2 (203–278 IU/L) and tertile 3 (>278 IU/L), compared with those in tertile 1 (<203 IU/L), among 972 patients with acute decompensated heart failure, and who were not on hemodialysis or peritoneal dialysis. Of note, this study was performed in patients with acute decompensated heart failure, whose pathophysiology differed from that of the general population. In addition, serum ALP levels in this study were considerably higher (median, 238 IU/L) than that of the current study (median, 92 IU/L). Another recent study (Majoni et al., 2020) including only 547 adults suggested that there was a positive association between serum ALP and increased risk of poor renal outcomes. It is worth noting that the study sample was relative small, and some imported variables such as BP levels or information of medications were not included in the regression models, and therefore could not draw an accurate conclusion.

In contrast to the prior studies, our study provides some new insights in the field. It is by far the first and largest study of its kind demonstrating a positive association between serum ALP and the risk of CKD progression during a treatment period of 4.4 years in general treated hypertensive adults. Moreover, we further found that the increased risk of CKD progression associated with higher serum ALP levels could be significantly reduced by folic acid treatment this risk was reduced by 47% with folic acid treatment.

The exact mechanisms by which higher serum ALP increases the risk of CKD progression, or by which folic acid treatment has higher efficacy on reducing the risk in this subgroup remains to be delineated. However, our findings are biologically plausible based on the available literature. It is well-known that oxidative stress (Coppolino et al., 2018), endothelial dysfunctions (Jourde-Chiche et al., 2019), and chronic inflammation (Mihai et al., 2018) play important roles in the pathophysiology of CKD development. Previous studies have suggested that elevated ALP is significantly associated with systemic inflammation (Cheung et al., 2008; Damera et al., 2011). ALP levels may, therefore, partially reflect inflammation of hepatic origin. Additionally, a study conducted in hypertensive individuals revealed that higher serum ALP increased the risk of endothelial dysfunction (Perticone et al., 2015). The possible mechanisms for this may be that ALP can reduce nitric oxide (NO) bioavailability by inhibiting tyrosine kinase activity into endothelial cells, leading to the consequent impairment of endothelial NO synthase function and the reduction of NO production (Schultz-Hector et al., 1993; Boo and Jo, 2003). Moreover, increased ALP levels may promote the production of reactive oxygen species and apoptosis (Perticone et al., 2015). Taken together, we hypothesized that oxidative stress, endothelial dysfunction, and chronic inflammation that may mediate the associations of ALP with increased risk of CKD.

On the other hand, possible antioxidant activities and anti-inflammatory properties of folic acid have been reported in previous studies (Joshi et al., 2001; Solini et al., 2006). Prior studies have demonstrated that folic acid can scavenge thiyl radicals, repair these thiols at physiological pH (Joshi et al., 2001), and reduce levels of interleukins (Solini et al., 2006). Moreover, folic acid also plays a crucial role in endothelium-protection (Stanhewicz and Kenney, 2017). Folic acid, in counteracting these detrimental effects, could therefore, significantly reduce the risk of CKD in a population with increased ALP levels. However, the detailed underlying mechanisms still need to be further investigated in future studies.

In our study, the increased risk of CKD progression associated with ALP levels and the beneficial effect of folic acid were mainly observed among those in quartile 4 of ALP levels. However, only in China, there were about 244.5 million of the adult population ≥18 years of age had hypertension (Wang et al., 2018). That is to say that about 61 million Chinese adults had increased CKD progression risk associated with higher ALP (quartile 4), and may benefit from folic acid supplementation. During about 4.5 years of folic acid supplementation (a safe and inexpensive treatment), about 67.1 thousand Chinese hypertensive patients may be exempt from the CKD progression risk. Therefore, our findings may be clinically meaningful.

Several potential limitations are worth mentioning. First, this is a post-hoc analysis. Although our current study adjusted for a broad array of covariates in the regression models, the possibility of residual confounding cannot be excluded. Second, creatinine was accessed only at baseline and the exit visit; more frequent measurements of creatinine would have allowed for a more accurate assessment of CKD progression. Third, in the current study, we collected total serum ALP rather than ALP isozymes. Fourth, our study was performed in Chinese hypertensive patients. The generalization of our results to other populations or patients with other kidney diseases needs to be further determined. Overall, further confirmation of our findings in more studies is essential.

In conclusions, our study suggests that higher serum ALP levels were associated with increased risk of CKD progression in general hypertensive adults, and this risk was reduced by 47% with folic acid treatment. Obtaining ALP levels is relatively easy, rapid, and universally available in general clinical laboratories. Our findings, if confirmed, have important clinical and public health implications. Our data suggest that identifying hypertensive patients with higher serum ALP levels, could help detect those individuals who are at high risk of CKD progression and who would particularly benefit from folic acid supplementation, a treatment that is simple, safe, and inexpensive.

## Data Availability

The raw data supporting the conclusions of this article will be made available by the authors, without undue reservation.
