# Supplementary material for: Interaction of Serum Alkaline Phosphatase and Folic Acid Treatment on Chronic Kidney Disease Progression in Treated Hypertensive Adults
Source: Front Pharmacol. 2022 Jan 13;12:753803. doi: 10.3389/fphar.2021.753803 (PMC8793861; doi:10.3389/fphar.2021.753803)
Supplement: Supplementary file 1 [file DataSheet1.docx]

**Interaction of serum alkaline phosphatase and folic acid treatment on chronic kidney disease progression in treated hypertensive adults**

**Supplemental Figure 1.** Flow chart of study participants

**Supplemental Figure 2.** The relation of baseline serum alkaline phosphatase with CKD incidence^†^ (A), rapid decline in renal function (B) and annual rate of relative decline in eGFR (C) in the enalapril-only population

**Supplemental Table 1.** Characteristics of the study participants by baseline serum alkaline phosphatase quartiles in the enalapril-only group

**Supplemental Table 2.** Characteristics of the study participants by baseline serum ALP quartiles among females in the enalapril-only group

**Supplemental Table 3.** Characteristics of the study participants by baseline serum alkaline phosphatase (ALP) quartiles among males in the enalapril-only group

**Supplemental Table 4.** The association between normal baseline serum ALP (20 to 140 IU/L) and renal outcomes in the enalapril-only population

**Supplemental Table 5.** The association between baseline serum ALP and CKD progression, with further adjustment for liver enzymes in the enalapril-only population

**Supplemental Table 6.** Effect modification of normal baseline serum ALP levels (20 to 140 IU/L) on folic acid treatment in renal outcomes in the total participants

**Supplemental Table 7.** Effect modification of baseline serum ALP levels (<110 and ≥110 IU/L) on folic acid treatment in CKD progression, with further adjustments for liver enzymes in the total participants

**Supplemental Table 8.** Effect modification of baseline serum ALP levels (<110 (Quartile 1-3) and ≥110 IU/L) on folic acid treatment in CKD progression in subgroups

15,104 participants enrolled in the Renal Sub-study

Excluded n=2,187;

Missing renal outcomes

12,917 participants analyzed for the renal outcome

Excluded n=183;

Missing alkaline phosphatase levels or with baseline liver diseases or missing data on liver diseases

12,734 participants included in final analyses

Enalapril-folic acid group n=6,345 (49.8%)

Enalapril-only group

n=6,389 (50.2%)

**Supplemental Figure 1. Flow chart of study participants**

**
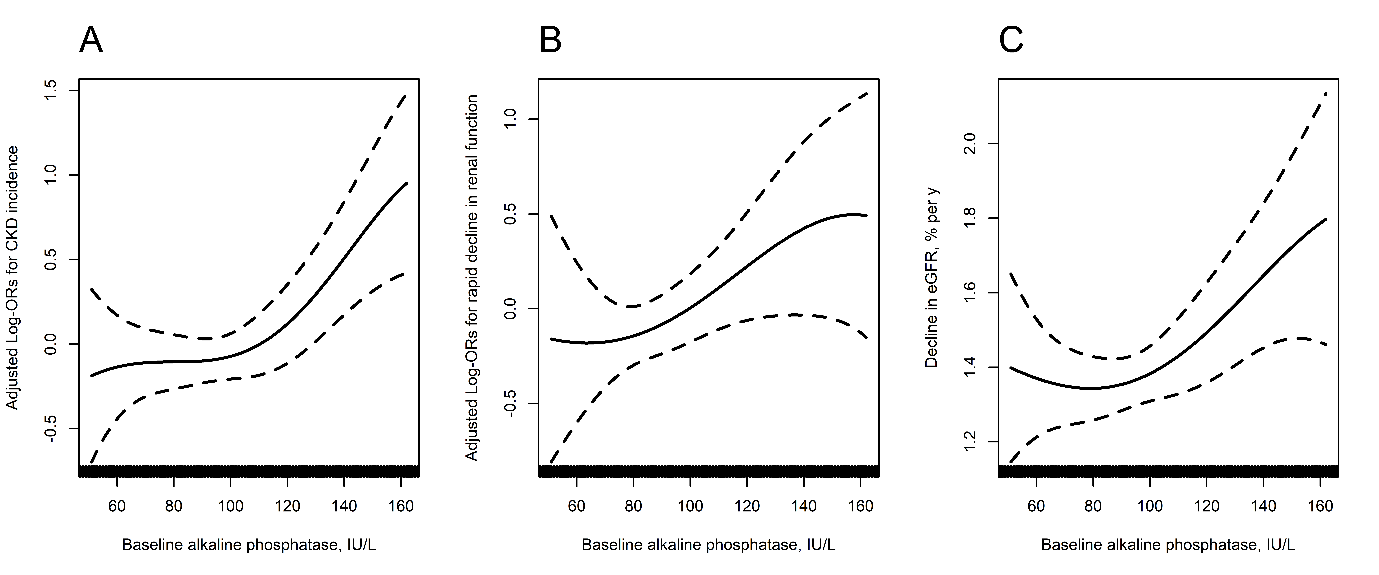
**

**Supplemental Figure 2. The relation of baseline serum alkaline phosphatase with CKD incidence** **^†^ (A), rapid decline in renal function (B) and annual rate of relative decline in eGFR (C) in the enalapril-only population^*^**

^*^Adjusted for age, sex, body mass index, smoking, alcohol drinking, albumin-corrected calcium, phosphate, uric acid, total cholesterol, fasting glucose, eGFR, systolic blood pressure (SBP), proteinuria and antihypertensive drug usage at baseline, as well as time-averaged SBP, the use of calcium channel blockers (CCB) and diuretics during the treatment period.

^†^Only participants with baseline eGFR ≥60mL/min/1.73m^2^ were included in this analysis.

**Abbreviations:** CKD, chronic kidney disease; eGFR, estimated glomerular filtration rate

**Supplemental Table 1. Characteristics of the study participants by baseline serum alkaline phosphatase quartiles in the enalapril-only group ^a^**

| Variables | Alkaline phosphatase quartiles, IU/L | | | | *P* value |
| --- | --- | --- | --- | --- | --- |
|  | Q1  <76 | Q2  76 to <92 | Q3  92 to <110 | Q4  ≥110 |  |
| **Baseline** |  |  |  |  |  |
| N | 1520 | 1671 | 1536 | 1662 |  |
| Male, No. (%) | 780 (51.3) | 751 (44.9) | 548 (35.7) | 402 (24.2) | <0.001 |
| Age, y | 57.8 ± 8.1 | 59.6 ± 7.6 | 60.3 ± 7.3 | 60.3 ± 6.7 | <0.001 |
| Body mass index, kg/m^2^ | 25.8 ± 3.5 | 25.6 ± 3.5 | 25.7 ± 3.6 | 25.6 ± 3.5 | 0.462 |
| SBP at baseline, mmHg | 165.9 ± 20.7 | 168.6 ± 21.2 | 169.5 ± 21.3 | 170.5 ± 21.0 | <0.001 |
| DBP at baseline, mmHg | 95.9 ± 11.9 | 95.3 ± 12.2 | 95.0 ± 12.0 | 94.8 ± 12.2 | 0.083 |
| Current smoking, No. (%) | 408 (26.8) | 416 (24.9) | 329 (21.4) | 263 (15.8) | <0.001 |
| Current alcohol drinking, No. (%) | 511 (33.6) | 435 (26.0) | 310 (20.2) | 215 (12.9) | <0.001 |
| Diabetes, No. (%) | 173 (11.4) | 196 (11.7) | 207 (13.5) | 308 (18.5) | <0.001 |
| Proteinuria, No. (%) | 114 (7.8) | 142 (8.8) | 150 (10.1) | 191 (12.0) | <0.001 |
| **Laboratory results** |  |  |  |  |  |
| Total cholesterol, mmol/L | 5.7 ± 1.2 | 5.8 ± 1.2 | 5.7 ± 1.1 | 5.7 ± 1.3 | 0.181 |
| HDL-C, mmol/L | 1.3 ± 0.4 | 1.3 ± 0.4 | 1.3 ± 0.3 | 1.4 ± 0.4 | 0.002 |
| Triglycerides, mmol/L | 1.5 ± 0.9 | 1.7 ± 1.0 | 1.7 ± 0.9 | 1.9 ± 1.0 | <0.001 |
| Fasting glucose, mmol/L | 5.9 ± 1.4 | 5.9 ± 1.4 | 6.0 ± 1.8 | 6.4 ± 2.4 | <0.001 |
| Uric acid, μmol/L | 296.6 ± 84.1 | 296.0 ± 75.1 | 292.9 ± 76.2 | 289.0 ± 78.2 | 0.024 |
| Phosphate, mmol/L | 1.2 ± 0.2 | 1.3 ± 0.2 | 1.3 ± 0.2 | 1.4 ± 0.3 | <0.001 |
| Albumin-corrected calcium, mmol/L | 2.4 ± 0.2 | 2.4 ± 0.1 | 2.5 ± 0.1 | 2.5 ± 0.2 | <0.001 |
| eGFR, mL/min1.73/m^2^ | 94.1 ± 13.1 | 93.8 ± 12.7 | 93.9 ± 12.3 | 94.9 ± 12.5 | 0.051 |
| Folate, ng/mL | 7.8 ± 3.2 | 7.6 ± 3.4 | 7.5 ± 3.0 | 7.9 ± 3.3 | 0.004 |
| Vitamin B_12_, pg/mL | 406.9 ± 152.9 | 411.9 ± 173.2 | 400.8 ± 148.6 | 410.6 ± 186.5 | 0.239 |
| **Medication use, No. (%)** |  |  |  |  |  |
| Antihypertensive drugs | 810 (53.3) | 820 (49.1) | 783 (51.0) | 794 (47.8) | 0.012 |
| Glucose-lowering drugs | 20 (1.3) | 23 (1.4) | 28 (1.8) | 34 (2.0) | 0.294 |
| Lipid-lowering drugs | 18 (1.2) | 20 (1.2) | 7 (0.5) | 11 (0.7) | 0.054 |
| Antiplatelet drugs | 77 (5.1) | 80 (4.8) | 59 (3.8) | 53 (3.2) | 0.030 |
| **During treatment period** |  |  |  |  |  |
| Time-averaged on-treatment SBP | 138.6 ± 10.4 | 139.8 ± 10.9 | 140.0 ± 11.5 | 140.1 ± 10.9 | <0.001 |
| Time-averaged on-treatment DBP | 84.4 ± 7.1 | 83.7 ± 7.4 | 83.2 ± 7.3 | 82.9 ± 7.5 | <0.001 |
| **Medication use, No. (%)** |  |  |  |  |  |
| Calcium channel blockers | 1222 (80.4) | 1368 (81.9) | 1247 (81.2) | 1356 (81.6) | 0.735 |
| Diuretics | 913 (60.1) | 1017 (60.9) | 951 (61.9) | 1005 (60.5) | 0.748 |
| Glucose-lowering drugs | 15 (1.0) | 26 (1.6) | 33 (2.1) | 39 (2.3) | 0.017 |
| Lipid-lowering drugs | 3 (0.2) | 0 (0.0) | 2 (0.1) | 0 (0.0) | 0.117 |
| Antiplatelet drugs | 14 (0.9) | 21 (1.3) | 15 (1.0) | 9 (0.5) | 0.193 |

^a^ Variables are presented as Mean ± SD or n (%)

**Abbreviations:** ALP, alkaline phosphatase; DBP, diastolic blood pressure; eGFR, estimated glomerular filtration rate; HDL-C, high-density lipoprotein; SBP, systolic blood pressure

**Supplemental Table 2. Characteristics of the study participants by baseline serum ALP quartiles among females in the enalapril-only group ^a^**

| **Variables** | **serum ALP, IU/L** | | | | ***P* value** |
| --- | --- | --- | --- | --- | --- |
|  | **<76** | **76 to <92** | **92 to <110** | **≥110** |  |
| **Baseline** |  |  |  |  |  |
| N | 740 | 920 | 988 | 1260 |  |
| Age, y | 55.7 ± 7.9 | 58.5 ± 7.6 | 60.1 ± 7.2 | 60.4 ± 6.4 | <0.001 |
| Body mass index, kg/m^2^ | 26.5 ± 3.6 | 26.0 ± 3.5 | 26.0 ± 3.7 | 25.8 ± 3.5 | 0.002 |
| SBP at baseline, mmHg | 165.8 ± 20.3 | 168.8 ± 20.9 | 170.4 ± 20.6 | 170.9 ± 20.9 | <0.001 |
| DBP at baseline, mmHg | 94.5 ± 10.7 | 93.5 ± 11.4 | 93.8 ± 11.8 | 93.9 ± 11.7 | 0.337 |
| Current smoking, No. (%) | 18 (2.4) | 34 (3.7) | 35 (3.5) | 49 (3.9) | 0.703 |
| Current alcohol drinking, No. (%) | 26 (3.5) | 41 (4.5) | 26 (2.6) | 38 (3.0) | 0.159 |
| Diabetes, No. (%) | 76 (10.3) | 106 (11.5) | 136 (13.8) | 239 (19.0) | <0.001 |
| Proteinuria, No. (%) | 53 (7.4) | 68 (7.7) | 93 (9.7) | 135 (11.1) | 0.012 |
| **Laboratory results** |  |  |  |  |  |
| Total cholesterol, mmol/L | 5.7 ± 1.2 | 5.8 ± 1.2 | 5.8 ± 1.2 | 5.8 ± 1.3 | 0.133 |
| HDL-C, mmol/L | 1.3 ± 0.3 | 1.3 ± 0.3 | 1.3 ± 0.3 | 1.4 ± 0.4 | <0.001 |
| Triglycerides, mmol/L | 1.6 ± 0.9 | 1.7 ± 0.9 | 1.8 ± 0.9 | 1.9 ± 1.0 | <0.001 |
| Fasting glucose, mmol/L | 5.8 ± 1.3 | 5.9 ± 1.4 | 6.1 ± 1.8 | 6.5 ± 2.4 | <0.001 |
| Uric acid, μmol/L | 260.6 ± 67.3 | 266.8 ± 63.8 | 272.0 ± 66.2 | 275.1 ± 71.8 | <0.001 |
| Phosphate, mmol/L | 1.3 ± 0.2 | 1.3 ± 0.2 | 1.4 ± 0.2 | 1.4 ± 0.2 | <0.001 |
| Albumin-corrected calcium, mmol/L | 2.4 ± 0.2 | 2.5 ± 0.1 | 2.5 ± 0.1 | 2.5 ± 0.2 | <0.001 |
| eGFR, mL/min1.73/m^2^ | 95.9 ± 13.5 | 94.5 ± 12.9 | 94.1 ± 12.4 | 95.0 ± 11.9 | 0.025 |
| Folate, ng/mL | 8.4 ± 3.1 | 8.2 ± 3.7 | 7.9 ± 3.0 | 8.2 ± 3.3 | 0.032 |
| Vitamin B12, pg/mL | 402.8 ± 139.5 | 416.3 ± 174.5 | 403.8 ± 137.4 | 414.3 ± 188.6 | 0.175 |
| **Medication use, No. (%)** |  |  |  |  |  |
| Antihypertensive drugs | 425 (57.4) | 473 (51.4) | 525 (53.1) | 609 (48.3) | 0.001 |
| Glucose-lowering drugs | 15 (2.0) | 15 (1.6) | 22 (2.2) | 31 (2.5) | 0.605 |
| Lipid-lowering drugs | 10 (1.4) | 9 (1.0) | 5 (0.5) | 9 (0.7) | 0.254 |
| Antiplatelet drugs | 35 (4.7) | 44 (4.8) | 34 (3.4) | 33 (2.6) | 0.024 |
| **During treatment period** |  |  |  |  |  |
| Time-averaged on-treatment SBP | 138.5 ± 10.7 | 139.6 ± 10.9 | 140.0 ± 11.7 | 139.9 ± 10.8 | 0.016 |
| Time-averaged on-treatment DBP | 84.0 ± 6.6 | 82.9 ± 6.9 | 82.3 ± 6.9 | 82.3 ± 6.9 | <0.001 |
| **Medication use, No. (%)** |  |  |  |  |  |
| Calcium channel blockers | 567 (76.6) | 726 (78.9) | 783 (79.3) | 1018 (80.8) | 0.174 |
| Diuretics | 416 (56.2) | 540 (58.7) | 598 (60.5) | 758 (60.2) | 0.259 |
| Glucose-lowering drugs | 7 (0.9) | 18 (2.0) | 25 (2.5) | 31 (2.5) | 0.084 |
| Lipid-lowering drugs | 0 (0.0) | 0 (0.0) | 2 (0.2) | 0 (0.0) | 0.116 |
| Antiplatelet drugs | 5 (0.7) | 8 (0.9) | 7 (0.7) | 4 (0.3) | 0.393 |

^a^ Variables are presented as Mean ± SD or n (%)

**Abbreviations:** ALP, alkaline phosphatase; DBP, diastolic blood pressure; eGFR, estimated glomerular filtration rate; HDL-C, high-density lipoprotein; SBP, systolic blood pressure

**Supplemental Table 3. Characteristics of the study participants by baseline serum alkaline phosphatase (ALP) quartiles among males in the enalapril-only group ^a^**

| **Variables** | **serum ALP, IU/L** | | | | ***P* value** |
| --- | --- | --- | --- | --- | --- |
|  | **<76** | **76 to <92** | **92 to <110** | **≥110** |  |
| **Baseline** |  |  |  |  |  |
| N | 780 | 751 | 548 | 402 |  |
| Age, y | 59.9 ± 7.8 | 61.0 ± 7.4 | 60.6 ± 7.5 | 60.0 ± 7.7 | 0.024 |
| Body mass index, kg/m^2^ | 25.1 ± 3.3 | 25.1 ± 3.3 | 25.1 ± 3.4 | 24.9 ± 3.5 | 0.559 |
| SBP at baseline, mmHg | 166.0 ± 21.0 | 168.5 ± 21.6 | 167.7 ± 22.3 | 169.1 ± 21.3 | 0.058 |
| DBP at baseline, mmHg | 97.2 ± 12.7 | 97.4 ± 12.9 | 97.2 ± 12.1 | 97.7 ± 13.4 | 0.928 |
| Current smoking, No. (%) | 390 (50.0) | 382 (50.9) | 294 (53.6) | 214 (53.2) | 0.814 |
| Current alcohol drinking, No. (%) | 485 (62.2) | 394 (52.5) | 284 (51.8) | 177 (44.0) | <0.001 |
| Diabetes, No. (%) | 97 (12.4) | 90 (12.0) | 71 (13.0) | 69 (17.2) | 0.075 |
| Proteinuria, No. (%) | 61 (8.2) | 74 (10.3) | 57 (10.8) | 56 (14.5) | 0.012 |
| **Laboratory results** |  |  |  |  |  |
| Total cholesterol, mmol/L | 5.6 ± 1.1 | 5.6 ± 1.2 | 5.5 ± 1.1 | 5.4 ± 1.1 | <0.001 |
| HDL-C, mmol/L | 1.4 ± 0.4 | 1.4 ± 0.4 | 1.3 ± 0.4 | 1.3 ± 0.4 | 0.581 |
| Triglycerides, mmol/L | 1.5 ± 0.9 | 1.7 ± 1.1 | 1.6 ± 0.9 | 1.8 ± 1.1 | <0.001 |
| Fasting glucose, mmol/L | 6.0 ± 1.5 | 5.9 ± 1.4 | 5.9 ± 1.6 | 6.1 ± 2.1 | 0.142 |
| Uric acid, μmol/L | 330.6 ± 84.3 | 331.7 ± 72.4 | 330.7 ± 78.4 | 332.8 ± 81.2 | 0.969 |
| Phosphate, mmol/L | 1.2 ± 0.2 | 1.2 ± 0.2 | 1.2 ± 0.2 | 1.3 ± 0.3 | <0.001 |
| Albumin-corrected calcium, mmol/L | 2.4 ± 0.2 | 2.4 ± 0.1 | 2.5 ± 0.1 | 2.5 ± 0.2 | <0.001 |
| eGFR, mL/min1.73/m^2^ | 92.5 ± 12.6 | 92.9 ± 12.4 | 93.6 ± 12.2 | 94.5 ± 14.2 | 0.055 |
| Folate, ng/mL | 7.2 ± 3.1 | 6.9 ± 2.9 | 6.6 ± 3.0 | 6.6 ± 3.1 | 0.004 |
| Vitamin B12, pg/mL | 410.7 ± 164.7 | 406.6 ± 171.6 | 395.4 ± 166.8 | 398.7 ± 179.5 | 0.367 |
| **Medication use, No. (%)** |  |  |  |  |  |
| Antihypertensive drugs | 385 (49.4) | 347 (46.2) | 258 (47.1) | 185 (46.0) | 0.581 |
| Glucose-lowering drugs | 5 (0.6) | 8 (1.1) | 6 (1.1) | 3 (0.7) | 0.761 |
| Lipid-lowering drugs | 8 (1.0) | 11 (1.5) | 2 (0.4) | 2 (0.5) | 0.162 |
| Antiplatelet drugs | 42 (5.4) | 36 (4.8) | 25 (4.6) | 20 (5.0) | 0.913 |
| **During treatment period** |  |  |  |  |  |
| Time-averaged on-treatment SBP | 138.8 ± 10.2 | 140.0 ± 11.0 | 139.9 ± 11.2 | 140.4 ± 11.2 | 0.044 |
| Time-averaged on-treatment DBP | 84.7 ± 7.4 | 84.6 ± 7.8 | 84.7 ± 7.6 | 85.0 ± 8.7 | 0.867 |
| **Medication use, No. (%)** |  |  |  |  |  |
| Calcium channel blockers | 655 (84.0) | 642 (85.5) | 464 (84.7) | 338 (84.1) | 0.856 |
| Diuretics | 497 (63.7) | 477 (63.5) | 353 (64.4) | 247 (61.4) | 0.815 |
| Glucose-lowering drugs | 8 (1.0) | 8 (1.1) | 8 (1.5) | 8 (2.0) | 0.494 |
| Lipid-lowering drugs | 3 (0.4) | 0 (0.0) | 0 (0.0) | 0 (0.0) | 0.088 |
| Antiplatelet drugs | 9 (1.2) | 13 (1.7) | 8 (1.5) | 5 (1.2) | 0.798 |

^a^ Variables are presented as Mean ± SD or n (%)

**Abbreviations:** ALP, alkaline phosphatase; DBP, diastolic blood pressure; eGFR, estimated glomerular filtration rate; HDL-C, high-density lipoprotein; SBP, systolic blood pressure

**Supplemental Table 4. The association between normal baseline serum ALP (20 to 140 IU/L) and renal outcomes in the enalapril-only population**

| **serum ALP, IU/L** | **N** | **No. of events (%)** | **Crude model** |  | **Adjusted model ^a^** |
| --- | --- | --- | --- | --- | --- |
|  |  |  | **OR (95% CI)** |  | **OR (95% CI)** |
| **CKD Progression** | |  |  |  |  |
| Categories |  |  |  |  |  |
| <110 | 4716 | 107 (2.3) | Ref. |  | Ref. |
| ≥110 | 1245 | 38 (3.1) | 1.36 (0.93, 1.97) |  | 1.42 (0.94, 2.14) |
| **CKD Incidence ^b^** | | |  |  |  |
| Categories |  |  |  |  |  |
| <110 | 4633 | 166 (3.6) | Ref. |  | Ref. |
| ≥110 | 1228 | 53 (4.3) | 1.21 (0.89, 1.66) |  | 1.29 (0.90, 1.87) |
| **Rapid decline in renal function** | | |  |  |  |
| Categories |  |  |  |  |  |
| <110 | 4715 | 67 (1.4) | Ref. |  | Ref. |
| ≥110 | 1245 | 21 (1.7) | 1.19 (0.73, 1.95) |  | 1.21 (0.71, 2.05) |
|  | N | Mean ± SD | β (95% CI) |  | β (95% CI) |
| **Decline in eGFR, % per y** | |  |  |  |  |
| Categories |  |  |  |  |  |
| <110 | 4715 | 1.3 ± 3.4 | Ref. |  | Ref. |
| ≥110 | 1245 | 1.7 ± 3.6 | 0.37 (0.15, 0.58) |  | 0.18 (-0.04, 0.39) |

^a^ Adjusted for age, sex, body mass index, smoking, alcohol drinking, albumin-corrected calcium, phosphate, uric acid, total cholesterol, fasting glucose, eGFR, systolic blood pressure (SBP), proteinuria and antihypertensive drug use at baseline, as well as time-averaged SBP, the use of calcium channel blockers (CCB) and diuretics during the treatment period.

^b^ Only participants with baseline eGFR ≥60mL/min/1.73m^2^ were included in this analysis.

**Abbreviations:** ALP, alkaline phosphatase; CI, confidence interval; CKD, chronic kidney disease; eGFR, estimated glomerular filtration rate; OR, odds ratio; SD, standard deviation

**Supplemental Table 5. The association between baseline serum ALP and CKD progression, with further adjustment for liver enzymes in the enalapril-only population**

| **serum ALP, IU/L** | **N** | **No. of events (%)** | **Model 1^a^** |  | **Model 2^b^** |
| --- | --- | --- | --- | --- | --- |
|  |  |  | **OR (95% CI)** |  | **OR (95% CI)** |
| **Quartiles** |  |  |  |  |  |
| Q1 (<76) | 1520 | 32 (2.1) | Ref. |  | Ref. |
| Q2 (76-<92) | 1671 | 37 (2.2) | 1.04 (0.63, 1.73) |  | 1.06 (0.64, 1.76) |
| Q3 (92-<110) | 1536 | 38 (2.5) | 0.96 (0.56, 1.62) |  | 0.99 (0.58, 1.67) |
| Q4 (≥110) | 1662 | 57 (3.4) | 1.60 (0.97, 2.65) |  | 1.70 (1.02, 2.82) |
| **Categories** |  |  |  |  |  |
| Q1-3 (<110) | 4727 | 107 (2.3) | Ref. |  | Ref. |
| Q4 (≥110) | 1662 | 57 (3.4) | 1.61 (1.11, 2.32) |  | 1.67 (1.16, 2.42) |

^a^ Model 1: Adjusted for age, sex, body mass index, smoking, alcohol drinking, albumin-corrected calcium, phosphate, uric acid, total cholesterol, fasting glucose, eGFR, systolic blood pressure (SBP), proteinuria and antihypertensive drug usage at baseline, as well as time-averaged SBP, the use of calcium channel blockers (CCB) and diuretics during the treatment period.

^b^ Model 2: Model 1 plus gamma glutamyl transpeptidase (GGT), alanine aminotransferase (ALT), aspartate aminotransferase (AST).

**Abbreviations:** ALP, alkaline phosphatase; CI, confidence interval; CKD, chronic kidney disease; eGFR, estimated glomerular filtration rate; OR, odds ratio

**Supplemental Table 6. Effect modification of normal baseline serum ALP levels (20 to 140 IU/L) on folic acid treatment in renal outcomes in the total participants**

| **serum ALP (IU/L)** | **Enalapril-only** | **Enalapril-**  **folic acid** | **Crude Model** | | **Adjusted Model ^a^** | |
| --- | --- | --- | --- | --- | --- | --- |
|  | **Events (%)** | **Events (%)** | **OR (95%CI)** | ***P*-interaction** | **OR (95%CI)** | ***P*-**  **interaction** |
| **CKD Progression** | |  |  | 0.089 |  | 0.036 |
| <110 | 107 (2.3) | 93 (2.0) | 0.88 (0.66, 1.16) |  | 0.90 (0.67, 1.21) |  |
| ≥110 | 38 (3.1) | 20 (1.6) | 0.52 (0.30, 0.90) |  | 0.46 (0.26, 0.81) |  |
| **CKD Incidence ^b^** | |  |  | 0.203 |  | 0.071 |
| <110 | 166 (3.6) | 151 (3.3) | 0.92 (0.74, 1.15) |  | 0.97 (0.75, 1.24) |  |
| ≥110 | 53 (4.3) | 36 (2.9) | 0.67 (0.44, 1.04) |  | 0.59 (0.36, 0.95) |  |
| **Rapid decline in renal function** | | |  | 0.327 |  | 0.150 |
| <110 | 67 (1.4) | 60 (1.3) | 0.91 (0.64, 1.29) |  | 0.94 (0.66, 1.36) |  |
| ≥110 | 21 (1.7) | 13 (1.0) | 0.62 (0.31, 1.24) |  | 0.53 (0.26, 1.08) |  |
|  | **Mean± SD** | **Mean± SD** | **β (95% CI)** | ***P*-interaction** | **β (95% CI)** | ***P*-interaction** |
| **Decline in eGFR, % per y** | | |  | 0.082 |  | 0.049 |
| <110 | 1.3 ± 3.4 | 1.2 ± 3.2 | -0.07 (-0.20, 0.07) |  | -0.06 (-0.19, 0.08) |  |
| ≥110 | 1.7 ± 3.6 | 1.3 ± 3.6 | -0.33 (-0.59, -0.07) |  | -0.34 (-0.60, -0.09) |  |

^a^ Adjusted for age, sex, body mass index, smoking, alcohol drinking, albumin-corrected calcium, phosphate, uric acid, total cholesterol, fasting glucose, eGFR, systolic blood pressure (SBP), proteinuria and antihypertensive drug usage at baseline, as well as time-averaged SBP, the use of calcium channel blockers (CCB) and diuretics during the treatment period.

^b^ Only participants with baseline eGFR ≥60mL/min/1.73m^2^ were included in this analysis.

**Abbreviations:** ALP, alkaline phosphatase; CI, confidence interval; CKD, chronic kidney disease; eGFR, estimated glomerular filtration rate; OR, odds ratio; SD, standard deviation

**Supplemental Table 7. Effect modification of baseline serum ALP levels** **(<110 and ≥110 IU/L) on folic acid treatment in CKD progression, with further adjustments for liver enzymes** **in the total participants**

| **serum ALP (IU/L)** | **Enalapril-only** | **Enalapril-**  **folic acid** | **Model 1^a^** | | **Model 2^b^** | |
| --- | --- | --- | --- | --- | --- | --- |
|  | **Events (%)** | **Events (%)** | **OR (95%CI)** | ***P*-interaction** | **OR (95%CI)** | ***P*-**  **interaction** |
| **Categories** | |  |  | 0.047 |  | 0.047 |
| <110 | 107 (2.3) | 93 (2.0) | 0.91 (0.68, 1.22) |  | 0.91 (0.68, 1.22) |  |
| ≥110 | 57 (3.4) | 36 (2.1) | 0.53 (0.34, 0.83) |  | 0.53 (0.34, 0.83) |  |

^a^ Model 1: Adjusted for age, sex, body mass index, smoking, alcohol drinking, albumin-corrected calcium, phosphate, uric acid, total cholesterol, fasting glucose, eGFR, systolic blood pressure (SBP), proteinuria and antihypertensive drug usage at baseline, as well as time-averaged SBP, the use of calcium channel blockers (CCB) and diuretics during the treatment period.

^b^ Model 2: Model 1 plus gamma glutamyl transpeptidase (GGT), alanine aminotransferase (ALT), aspartate aminotransferase (AST).

**Abbreviations:** ALP, alkaline phosphatase; CI, confidence interval; CKD, chronic kidney disease; eGFR, estimated glomerular filtration rate; OR, odds ratio

**Supplemental Table 8. Effect modification of baseline serum ALP levels (<110 (Quartile 1-3) and ≥110 IU/L) on folic acid treatment in CKD progression in subgroups**

| **Serum ALP (IU/L)** | **Enalapril-only** | **Enalapril-**  **folic acid** | **Adjusted Model ^a^** | |
| --- | --- | --- | --- | --- |
|  | **Events (%)** | **Events (%)** | **OR (95%CI)** | ***P*-interaction** |
| **Male** |  |  |  | 0.692 |
| <110 | 53 (2.5) | 36 (1.8) | 0.67 (0.42, 1.04) |  |
| ≥110 | 14 (3.5) | 9 (2.3) | 0.55 (0.23, 1.32) |  |
| **Female** |  |  |  | 0.020 |
| <110 | 54 (2.0) | 57 (2.1) | 1.14 (0.77, 1.68) |  |
| ≥110 | 43 (3.4) | 27 (2.1) | 0.53 (0.32, 0.89) |  |
| **Age <65, y** |  |  |  | 0.408 |
| <110 | 42 (1.2) | 37 (1.1) | 0.98 (0.62, 1.56) |  |
| ≥110 | 26 (2.1) | 20 (1.6) | 0.71 (0.38, 1.31) |  |
| **Age ≥65** |  |  |  | 0.036 |
| <110 | 65 (5.3) | 56 (4.7) | 0.89 (0.61, 1.31) |  |
| ≥110 | 31 (7.4) | 16 (3.4) | 0.41 (0.21, 0.78) |  |
| **BMI <24, kg/m^2^** |  |  |  | 0.579 |
| <110 | 37 (2.4) | 31 (2.0) | 0.83 (0.50, 1.39) |  |
| ≥110 | 17 (3.0) | 11 (2.1) | 0.64 (0.29, 1.41) |  |
| **BMI ≥24** |  |  |  | 0.043 |
| <110 | 70 (2.2) | 62 (2.0) | 0.95 (0.66, 1.36) |  |
| ≥110 | 40 (3.7) | 25 (2.2) | 0.49 (0.29, 0.84) |  |
| **Baseline SBP <160, mmHg** | |  |  | 0.227 |
| <110 | 24 (1.4) | 27 (1.6) | 1.17 (0.66, 2.07) |  |
| ≥110 | 14 (2.7) | 11 (2.0) | 0.63 (0.27, 1.45) |  |
| **Baseline SBP ≥160** | |  |  | 0.108 |
| <110 | 83 (2.7) | 66 (2.2) | 0.83 (0.59, 1.17) |  |
| ≥110 | 43 (3.7) | 25 (2.2) | 0.50 (0.30, 0.84) |  |
| **Time-averaged SBP <140, mmHg** | | |  | 0.249 |
| <110 | 47 (1.8) | 44 (1.6) | 0.92 (0.60, 1.41) |  |
| ≥110 | 24 (2.8) | 17 (1.9) | 0.58 (0.30, 1.11) |  |
| **Time-averaged SBP ≥140** | | |  | 0.107 |
| <110 | 60 (2.9) | 49 (2.5) | 0.92 (0.61, 1.38) |  |
| ≥110 | 33 (4.2) | 19 (2.4) | 0.51 (0.28, 0.93) |  |
| **Baseline TC <5.2, mmol/L** | |  |  | 0.537 |
| <110 | 41 (2.5) | 30 (1.9) | 0.71 (0.43, 1.16) |  |
| ≥110 | 20 (3.4) | 12 (2.2) | 0.53 (0.25, 1.14) |  |
| **Baseline TC ≥5.2** | |  |  | 0.050 |
| <110 | 66 (2.1) | 63 (2.1) | 1.04 (0.72, 1.51) |  |
| ≥110 | 37 (3.4) | 24 (2.1) | 0.54 (0.32, 0.94) |  |
| **Total homocysteine <12.5,** **μmol/L (median)** | | |  | 0.375 |
| <110 | 31 (1.3) | 27 (1.2) | 0.87 (0.51, 1.49) |  |
| ≥110 | 20 (2.3) | 12 (1.4) | 0.58 (0.28, 1.21) |  |
| **Total homocysteine** ≥**12.5** | |  |  | 0.109 |
| <110 | 76 (3.2) | 66 (2.8) | 0.92 (0.65, 1.31) |  |
| ≥110 | 36 (4.6) | 24 (2.8) | 0.54 (0.31, 0.94) |  |
| **Folate <7.3, ng/mL (median)** | |  |  | 0.265 |
| <110 | 61 (2.6) | 57 (2.4) | 0.91 (0.62, 1.33) |  |
| ≥110 | 25 (3.2) | 19 (2.4) | 0.59 (0.31, 1.13) |  |
| **Folate** ≥**7.3** |  |  |  | 0.234 |
| <110 | 45 (2.0) | 34 (1.5) | 0.87 (0.54, 1.40) |  |
| ≥110 | 30 (3.5) | 17 (2.0) | 0.54 (0.29, 1.01) |  |
| **Vitamin B12 <****370.8, pg/mL (median)** | |  |  | 0.189 |
| <110 | 57 (2.4) | 54 (2.3) | 1.01 (0.68, 1.50) |  |
| ≥110 | 27 (3.3) | 18 (2.1) | 0.61 (0.33, 1.15) |  |
| **Vitamin B12** ≥**370.8** |  |  |  | 0.335 |
| <110 | 49 (2.1) | 37 (1.6) | 0.78 (0.50, 1.22) |  |
| ≥110 | 28 (3.4) | 18 (2.2) | 0.53 (0.28, 1.01) |  |
| **Phosphate <1.3, mmol/L (median)** | |  |  | 0.031 |
| <110 | 64 (2.5) | 49 (2.0) | 0.82 (0.55, 1.21) |  |
| ≥110 | 22 (3.7) | 7 (1.2) | 0.30 (0.12, 0.71) |  |
| **Phosphate** ≥**1.3** |  |  |  | 0.217 |
| <110 | 43 (2.0) | 44 (2.0) | 1.04 (0.67, 1.62) |  |
| ≥110 | 35 (3.3) | 29 (2.6) | 0.68 (0.40, 1.14) |  |
| **Albumin-corrected calcium <2.4, mmol/L** **(median)** | |  |  | 0.186 |
| <110 | 56 (2.2) | 46 (1.8) | 0.78 (0.52, 1.18) |  |
| ≥110 | 20 (3.2) | 10 (1.6) | 0.43 (0.20, 0.95) |  |
| **Albumin-corrected calcium** ≥**2.4** | |  |  | 0.096 |
| <110 | 51 (2.4) | 47 (2.2) | 1.08 (0.71, 1.64) |  |
| ≥110 | 37 (3.6) | 26 (2.5) | 0.61 (0.36, 1.04) |  |
| **FG <5.6 mmol/L** |  |  |  | 0.118 |
| <110 | 52 (2.3) | 50 (2.2) | 1.01 (0.67, 1.52) |  |
| ≥110 | 28 (3.7) | 18 (2.2) | 0.56 (0.30, 1.04) |  |
| **FG** ≥**5.6 mmol/L or diabetes ^b^** | |  |  | 0.186 |
| <110 | 55 (2.2) | 43 (1.8) | 0.82 (0.54, 1.25) |  |
| ≥110 | 29 (3.2) | 18 (2.0) | 0.49 (0.26, 0.93) |  |
| **No CKD** | |  |  | 0.248 |
| <110 | 76 (1.9) | 73 (1.8) | 1.03 (0.74, 1.43) |  |
| ≥110 | 34 (2.4) | 27 (1.9) | 0.72 (0.43, 1.21) |  |
| **CKD ^c^** | |  |  | 0.144 |
| <110 | 24 (5.2) | 17 (3.4) | 0.60 (0.31, 1.14) |  |
| ≥110 | 22 (10.6) | 7 (3.3) | 0.27 (0.11, 0.65) |  |
| **No diuretics usage during the treatment period** | | |  | 0.242 |
| <110 | 30 (1.6) | 27 (1.5) | 1.04 (0.59, 1.82) |  |
| ≥110 | 16 (2.4) | 10 (1.7) | 0.57 (0.25, 1.32) |  |
| **Diuretics usage during the treatment period** | | |  | 0.113 |
| <110 | 77 (2.7) | 66 (2.3) | 0.88 (0.62, 1.24) |  |
| ≥110 | 41 (4.1) | 26 (2.4) | 0.53 (0.32, 0.89) |  |
| **No CCB usage during the treatment period** | | |  | 0.003 |
| <110 | 15 (1.7) | 11 (1.3) | 0.93 (0.39, 2.21) |  |
| ≥110 | 14 (4.6) | 1 (0.3) | 0.06 (0.01, 0.47) |  |
| **CCB usage during the treatment period** | | |  | 0.274 |
| <110 | 92 (2.4) | 82 (2.2) | 0.93 (0.68, 1.28) |  |
| ≥110 | 43 (3.2) | 35 (2.5) | 0.68 (0.43, 1.09) |  |

^a^ Adjusted for age, sex, body mass index, smoking, alcohol drinking, albumin-corrected calcium, phosphate, uric acid, total cholesterol, fasting glucose, eGFR, systolic blood pressure (SBP), proteinuria and antihypertensive drug usage at baseline, as well as time-averaged SBP, the use of calcium channel blockers (CCB) and diuretics during the treatment period, if not stratified.

^b^ Diabetes was defined as having a history of diabetes, or under glucose-lowering therapy, or a fasting glucose ≥7mmol/L at baseline.

^c^ CKD was defined as having or proteinuria or an eGFR <60 mL/min/1.73m^2^ at baseline.

**Abbreviations:** ALP, alkaline phosphatase; BMI, body mass index; CI, confidence interval; CKD, chronic kidney disease; eGFR, estimated glomerular filtration rate; FG, fasting glucose; OR, odds ratio; SD, standard deviation; TC, total cholesterol
